# Glucocorticoid use among patients with rheumatoid arthritis during the first year of treatment—a cohort study

**DOI:** 10.1007/s00296-026-06186-1

**Published:** 2026-06-12

**Authors:** Kirsten S. Duch, Maiada Alkattan, Tóri Ásbjørn Matras Holm, Peremisan Pakyanathan, Lene Wohlfahrt Dreyer, Martin Bøgsted, Søren Lundbye-Christensen, Salome Kristensen

**Affiliations:** 1 Center of Rheumatic Research Aalborg (CERRA), Department of Rheumatology, Aalborg University Hospital, Aalborg, Denmark; 2 Research Data and Biostatistics, Aalborg University Hospital, Aalborg, Denmark; 3 Department of Clinical Medicine, Aalborg University, Aalborg, Denmark; 4 The DANBIO Registry, Glostrup, Denmark; 5 Center for Clinical Data Science (CLINDA), Aalborg University and Aalborg university hospital, Aalborg, Denmark

**Keywords:** Glucocorticoids, Arthritis rheumatoid, Infections, Registries

## Abstract

Glucocorticoid treatment for patients with rheumatoid arthritis (RA) is widely used as fast-acting bridging therapy to reduce disease activity. However, the safety is still discussed. This cohort study describes glucocorticoid dosage during the first year after treatment initiation in patients with RA and secondarily presents the one-year dosedependent infection risk. Hospital records were reviewed for 574 newly diagnosed RA cases treated at Department of Rheumatology Aalborg University Hospital between 2014 and 2022 to identify physician prescribed daily glucocorticoid dose and infections within the year following first visit. Daily glucocorticoid dose was presented. Secondary, the relative risk (RR) for one-year dose-dependent (non-exposed, low dose: 0-10 mg/day, high dose: >10 mg/day, from 21 days average) risk of first infection was calculated using modified Poisson regression. In total, 428 patients (75%) received glucocorticoid during the first year, with 313 (55%) initiated at first visit. Mean prescribed glucocorticoid dose was 9.3 mg/day, with 262 patients (46%) receiving tablets and 270 (47%) receiving intra-articular injection. Overall, 117 patients (20%) experienced an infection within the first year. The model was adjusted for asthma/chronic obstructive pulmonary disease, sex, and age, yielding a RR of 1.13 (95% confidence interval (CI): 0.77 to 1.67) for low-dose exposure versus non-exposure. Glucocorticoid dosages were highest early in disease course and declined substantially after the initial visit, suggesting that physicians in the Danish healthcare decrease dose within the first year after diagnosis. CI for dose-dependent infections risk were compatible with both benefit and harm.

**Supplementary Information:**

The online version contains supplementary material available at 10.1007/s00296-026-06186-1.

## Introduction

Rheumatoid arthritis (RA) is a chronic autoimmune disease affecting about 1% of the global population [1], with a Danish incidence of 35.5 per 100,000 person-years [2]. According to EULAR’s treatment recommendations, patients diagnosed with RA should begin treatment with disease modifying-antirheumatic drugs (DMARDs), as soon the diagnosis is established, combined with short-term glucocorticoids depending on disease activity [3].

The use of glucocorticoid therapy varies depending on the index date and population. In the QUEST-RA study from 2007, the use of prednisolone was assessed in 15 different countries, revealing a percentage of prednisolone users between 26% and 88% across the 15 countries, with an overall estimate of 66%, and 43% users in Denmark [4]. This study likely included more prevalent cases, as the mean disease duration was presented to be 11.5 years across all 15 countries. For comparison, a separate study examining opioid use in RA, indicated that depending on the duration of use, the calendar year, and geographical location, the percentage of users varied between 2% and 84% [5]. In a Danish study investigating mortality in RA, the proportion of patients exposed to glucocorticoid before the index date ranged from 22.8% to 30.6% depending on the calendar year of inclusion [6].

Observational studies in patients with RA have reported an increased risk of infections associated with glucocorticoid therapy, frequently with evidence of a dose-dependent relationship. In these studies, glucocorticoid exposure is typically assessed within predefined time periods before an index date and is most often based on prescription data, which may not accurately reflect the actual dose or dynamic dose adjustments over time [6–10]. In a registry-based cohort study by George et al. (2020), 247,297 patients with RA, receiving stable DMARD therapy, reported a dose-dependent increased risk of infections requiring hospitalization, with a statistically significant increased risk observed even at daily doses > 0 to ≤ 5 mg of prednisolone compared with no glucocorticoid use [10]. Roubile et al. (2021) followed a cohort of 608 patients over 10 years and reported a significantly higher frequency of infections among patients who received glucocorticoid at any time during follow-up compared with those never treated [11]. More recently, Barbulescu et al. (2023) included 9,654 newly diagnosed RA with up to three years of follow-up and found a dose-dependent increase in infection risk using models accounting for time-varying exposure and confounding [8]. Evidence from randomized controlled trials also suggests increased infection risk: a systematic review by Palmowski et al. (2023) including six trials with at least two years of follow-up reported higher infection risk among patients treated with long-term low-dose glucocorticoid (≤ 7.5 mg/day prednisone) compared with placebo (RR 1.40 (95% CI 1.19 to 1.65) [12]. However, the GLORIA trial suggested that despite an increased risk of infections long-term benefit and low risk of severe adverse events suggested a favour of low dose add on therapy [9].

The large variation in reported glucocorticoid use across studies may be due to differences in the definition of exposure, as well as variation in age, disease duration, and disease severity. Thus, the aim of this study was to present the daily glucocorticoid use among Danish patients with RA according to patient records and medical files the first year after diagnosis, using physician-reported exposure. A secondary aim was to present the observed infection risk following glucocorticoid treatment among the included patients.

## Methods

### Study population and data collection

This cohort study identified a total of 600 newly diagnosed adult (≥ 18 years) patients registered with an RA diagnosis (International classification of diseases, 10th version (ICD-10) code M05 or M06, excluding M06.1) registered in the Danish Clinical Quality Registry, DANBIO [13, 14] between 2014 and November 2022, treated at Aalborg University Hospital in Denmark. Registration of patients with RA in the DANBIO registry has been mandatory for all patients treated at a public hospital or private clinic since 2006 [13, 14]. The date of first registered visit in DANBIO was used as the index date (baseline). To ensure that the cohort reflected contemporary treatment practices, inclusion was restricted to patients with a first registered visit from 2014 onwards.

**Fig. 1:**
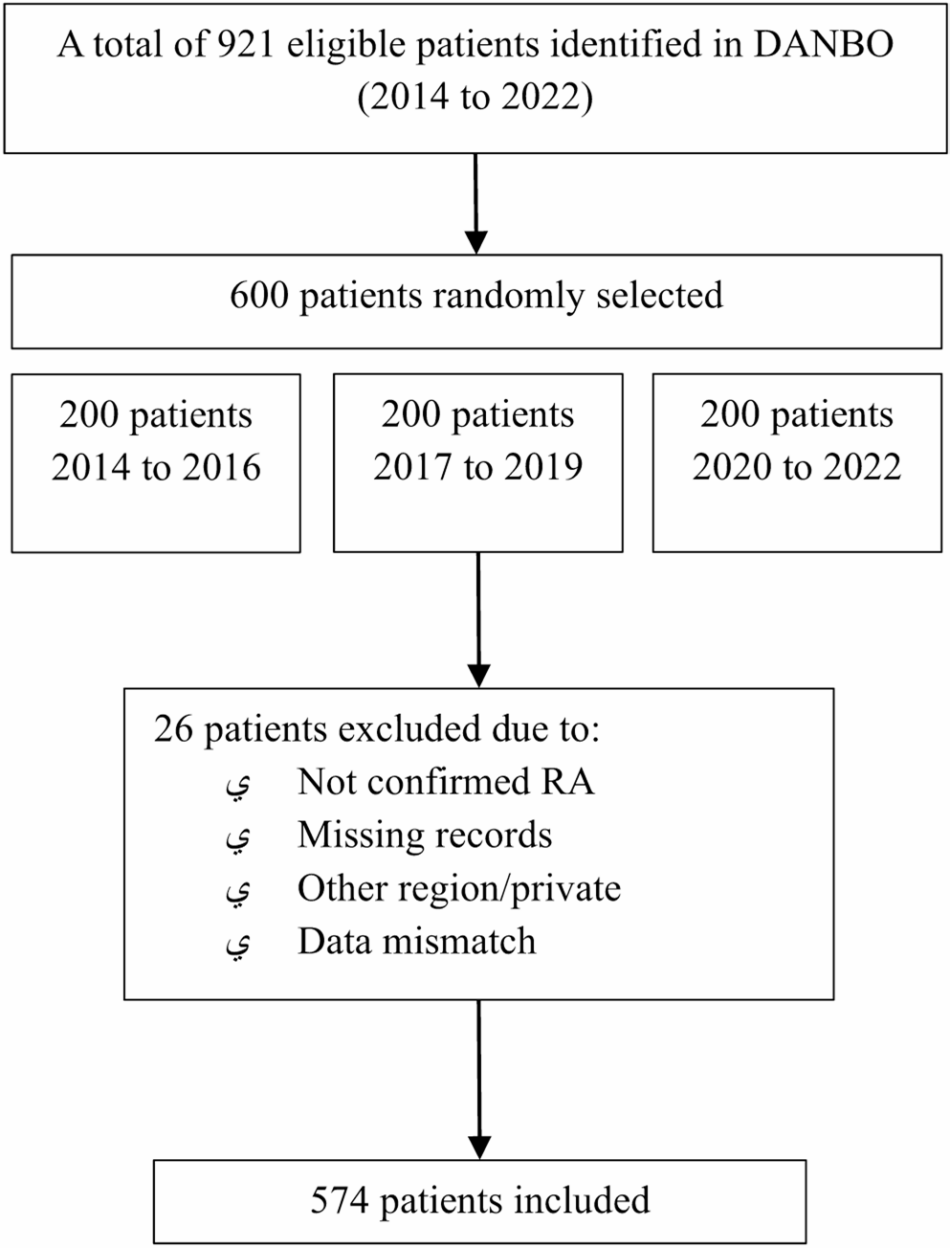
Flowchart

The 600 patients were randomly sampled from 921 eligible patients, with 200 patients registered with a first visit in each of the intervals 2014 to 2016, 2017 to 2019, and 2020 to November 2022, as differences through time may exist. Patients were excluded if their hospital records indicated only an initial, unconfirmed RA diagnosis, or if discrepancies between registry data and hospital records indicated that the date registered as the first visit in DANBIO did not correspond to the actual first rheumatology consultation (Fig. 1). Patients who died, were followed for infection and included until date of death. The sample size was chosen to be sufficiently large to present glucocorticoid use.

For the 574 included patients, data were collected from electronic patient hospital records. Collected data included daily glucocorticoid use (injections or tablets) within the year following first visit at the outpatient rheumatologic clinic, information on 28-joint disease activity score with C-reactive protein (DAS28-CRP) at first visit (from DANBIO records), date of disease onset, and relevant comorbidities. Finally, all infections requiring antibiotic treatment were recorded if they were the first infection within one year after the initial visit to the rheumatology outpatient clinic. Infections were classified based on patient records, and where specific disease codes were absent, the ICD-10 code most consistent with the description was applied. If the tapering regime of oral glucocorticoid was not specified in the records, a weekly tapering by 5 mg was assumed. Data were manually entered in dedicated electronic case report form in the Research Electronic Data Capture (REDCap) database hosted by Aalborg University hospital.

The following administration forms of glucocorticoid were recorded: Peroral, intraarticular, intravenous, intramuscular, whereas topical and inhalation were not registered. The equivalent prednisolone dosages were calculated.

### Statistics

To illustrate the daily glucocorticoid use from index date to one year following first visit at a rheumatologist, two daily averages of prednisolone-equivalent dosages were calculated [15]. Firstly, average glucocorticoid use per patients receiving glucocorticoid therapy on a given day, was calculated. Secondly, the average glucocorticoid use per patients (including patients not currently receiving any glucocorticoid treatment) was calculated. Both averages were presented graphically.

The number of first infections and observed risk within the first year was reported together with the one-year incidence rate for first infection.

For the secondary analysis with dose-dependent initial glucocorticoid use, the first 21 days after the first rheumatology visit was used to establish the initial glucocorticoid exposure. Specifically, the daily physician prescribed initial glucocorticoid dosage during the first 21 days were used to calculate the daily average during the first 21 days (with injection dose assumed given on the date the injection was given), which was in turn used to determine exposure group. This meant that for the dose-dependent analysis the one-year infections risk and incidence rate for first infection, was calculated for 344 days (corresponding to day 22 to day 365 after first rheumatology visit) based on initial glucocorticoid exposure. Patients with an infection within the first 21 days were excluded from the dose-dependent analysis. The relative risk (RR) of first infection within one year, was estimated using modified Poisson regression, crude, adjusted for chronic obstructive pulmonary disease (COPD) or asthma (binary), sex (binary), and age (continuous), and a complete case analysis adjusted for COPD or asthma (binary), sex (binary), age (continuous), and DAS28-CRP (continuous) [16]. For the RR estimation and initial glucocorticoid dose stratification, the first 21 days after the first visit were used to define the average initial glucocorticoid exposure groups in non-exposed, low/medium exposure (0–10 mg/day) and high exposure (> 10 mg/day).

A sensitivity analysis was conducted to avoid inclusion of prevalent cases. All models were repeated after excluding patients with more than 6 months between date of diagnose and index date. The 6-month cut-off was chosen to represent a reasonable time interval between diagnosis, and first rheumatology visit and to reduce the risk of including patients with delayed or retrospective registry entries. Results from the sensitivity analyses were presented in Supplementary material.

Estimates were presented with 95% confidence intervals (CI). Analysis and data management were performed in R version 4.2.2. Missing values were only relevant for the Poisson regression adjusting for DAS28-CRP and were handled as complete case analysis. The STROBE standard was used as guideline to ensure relevant information was included in the article (checklist added as supplementary material) [18] .

### Ethics and data availability

The study was based on medical files and DANBIO data and did not involve patients directly and adhere to the Declaration of Helsinki. Permission to access hospital records without informed consent was obtained from the North Denmark Region and the study was in accordance with the General Data Protection Regulation (GDPR) and Danish legislation (ID-nr. 2023–017371) prospectively registered 17 May 2023. Due to personal sensitive data, it was not possibly to publish the data set. According to Danish law, no ethical approval or informed consent from the patients was required for the current study.

Figure 1 describes the flowchart of the population from the total eligible individuals 921 to the 574 included patients.

## Results

Of the 600 sampled patients, 574 (96%) were included in the study (Fig. 1), of these, 145 (25%) had more than 6 months between the first registered visit and the diagnosis date and were excluded in the sensitivity analysis. Among the 574 included patients, 16 (3%) had an infection before or on day 21 and were not included in the dose-dependent analysis. The mean age ranged from 50.5 to 63.7 years across exposure groups, with a slightly higher mean age in the high-exposure group. The percentage of women was highest in the non-exposed group (68%) and lowest in the high-exposure group (62%). DAS28-CRP and the percentage of patients with COPD was highest among the high-exposure group (Table 1). Patients with a duration up to 6 months between first visit and diagnose did not differ substantially from the rest of the cohort (Table S1). Interestingly, patients with first visit in later years, were more likely to receive initial glucocorticoid therapy within the first 21 days (2014 to 2016 (nonexposed: 97/181 (54%), low dose: 69/181 (38%), high dose: 15/181 (8%)) vs. 2017 to 2019 (nonexposed: 62/185 (34%), low dose 98/185 (53%), high dose: 78/185 (42%)) vs. 2020 to 2022 (nonexposed: 47/192 (25%), low dose: 107/192 (56%), high dose: 38/192 (40%)).

**Table 1:** Patient characteristics at index for all and by glucocorticoid dose

		Nonexposed (*n* = 206)	Low dose (*n* = 274)	High dose (*n* = 78)	All^a^ (*n* = 574)
Age, mean years (sd)		50.5 (15.0)	62.4 (15.1)	63.7 (16.3)	62.2 (15.2)
Sex (women), n (%)		140 (68.0)	180 (65.7)	48 (61.5)	380 (66.2)
DAS28-CRP, mean (sd)		3.3 (1.4)	4.7 (1.1)	5.6 (0.9)	4.3 (1.4)
Missing, (%)		53 (25.7)	60 (22.0)	22 (28.0)	143 (25.0)
COPD, n (%)		11 (5.3)	27 (9.9)	10 (12.8)	52 (9.1)
Asthma, n (%)		22 (10.7)	23 (8.4)	6 (7.7)	56 (9.8)
Year of first visit, n (%)					
2014 to 2016		97 (47.1)	69 (25.2)	15 (19.2)	185 (32.2)
2017 to 2019		62 (30.1)	98 (35.8)	25 (32.1)	192 (33.4)
2020 to 2022		47 (22.8)	107 (39.1)	38 (48.7)	197 (34.3)

*sd* standard deviation, *n*:number, *COPD* chronic obstructive pulmonary disease, *DAS28-CRP* 28 joint disease activity score with c-reactive protein

^a^The total population includes all individuals from date of first visit, whereas the groups include the population from 21 days after first visit. Patients with an infection or lack of follow-up within the first 21 days will occur in the total but not in the dose stratification

**Fig. 2:**
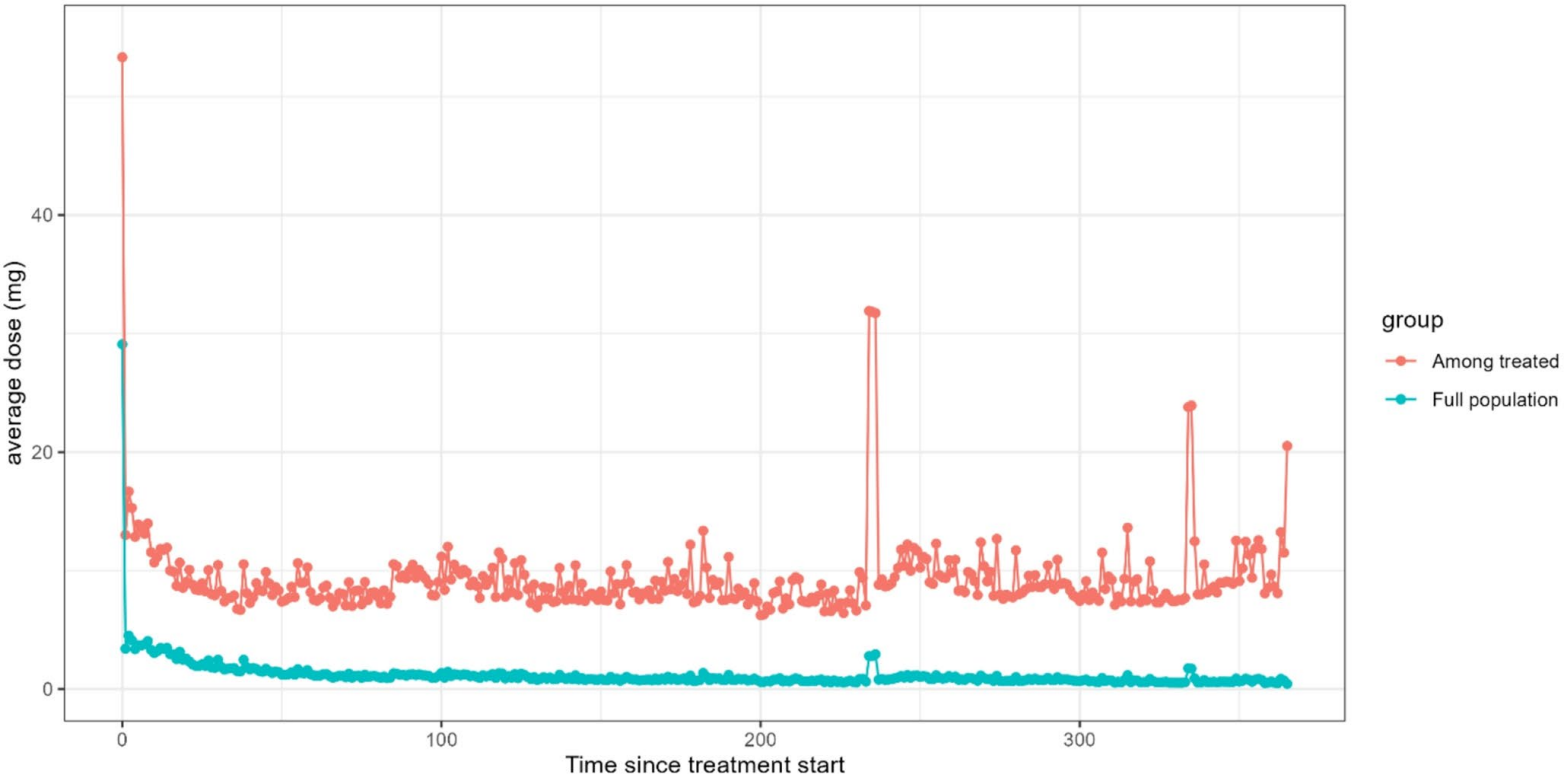
Observed daily glucocorticoid dose converted to prednisolone-equivalent dose

A total of 428 patients (75%) received glucocorticoid therapy at some point during the first year. The average glucocorticoid dose for the full population peaked on the day of treatment initiation, when 313 patients (55%) were treated, with an average dose of 29.1 mg. This average dropped to less than 5 mg/day during the remaining days of the year (Fig. 2), with an overall average of 1.2 mg/day for the whole year. Twelve (2%) patients were receiving treatment on day 365. Among treated patients, the average dose was 9.3 mg/day for the whole year, with a peak at the first visit (53.3 mg) and 234 days after the first visit (31.9 mg). The number of treated individuals at different time points is shown in supplementary Table S2. In the sensitivity analysis excluding patients with more than 6 months between first visit and date of diagnosis, results were similar, with an average dose of 0.9 mg/day for the full population and 11.0 mg/day among treated patients. The corresponding figure is presented in supplementary Figure S1.

The most common glucocorticoid administration routes were oral and intra-articular injections, with 262 patients (46%) receiving oral tablets and 270 (47%) receiving at least one intra-articular injection during the year (supplementary Table S3).

During the one-year follow-up, 117 patients (20%) experienced at least one infection, with the most common type of infections being respiratory infections or urinary tract infections. The one-year incidence rate was 0.24. Of the total number of infections, 101 occurred after day 21, which defined the exposure period. The incidence rates were higher in both initial glucocorticoid-exposed groups compared with the non-exposed reference group (Table 2).

Both crude and adjusted RRs indicated a higher infection risk among initial glucocorticoid-exposed patients compared with the non-exposed group (Table 2). However, the 95% CIs for both the low/medium and the high exposure groups showed results compatible with both benefit and harm. Combining the high and low exposure group did not change the results (crude RR: 1.21 (95% CI 0.83 to 1.76), adjusted RR: 1.12 (95% CI 0.77 to 1.62), adjusted RR including DAS28-CRP: 1.18 (0.69 to 2.02)). Excluding patients with more than 6 months between the first registered visit and diagnosis did not alter the results (supplementary Table S4 and S5).

Figure 2 describes the daily average glucocorticoid use the first year after first visit. The red line illustrates usage among treated, whereas the blue line illustrates average use among the full population.

**Table 2:** Infection risk, for the whole period and stratified on dosage based on first 21 days of treatment

		Nonexposed (*n* = 206)	Low/Medium dose (*n* = 274)	High dose (*n* = 78)	Total^a^ (*n* = 574)
*Observed values*					
Infections		33	53	15	117
Person years		187	241	68	493
Incidence rate per person year		0.18	0.22	0.22	0.24
Absolute risk		0.16	0.19	0.19	0.20
*Estimates from Poisson regression*					
Crude RR		1 (ref.)	1.20 (0.69 to 2.08)	1.16 (0.67 to 1.99)	-
Adjusted RR^b^		1 (ref.)	1.13 (0.77 to 1.67)	1.07 (0.64 to 1.82)	-
Complete case disease-activity adjusted RR^c^		1 (ref.)	1.20 (0.70 to 2.05)	1.01 (0.47 to 2.19)	-

*n* number, *ref* reference, *RR* relative risk, *COPD* chronic obstructive pulmonary disease, *DAS28-CRP* 28 joint disease activity score with c-reactive protein

^a^The total population includes all individuals from date of first visit, whereas the groups on dose include the population from 21 days after first visit. Patients with an infection or lack of follow-up within the first 21 days will occur in the total but not in the dose stratification

^b^The adjusted RR were adjusted for asthma or COPD, sex, and age

^c^The complete case adjusted RR were adjusted for asthma or COPD, sex, age, and DAS28-CRP

## Discussion

This study found that glucocorticoid use was common and highest at treatment initiation, followed by consistent tapering during the first year, while the observed infection risk demonstrated to wide confidence intervals to indicate any dose-dependent association.

The average glucocorticoid dose was highest during the initial phase of treatment, however despite individual fluctuations, the population average was quite stable during the rest of the year (Fig. 2). Similarly, the daily glucocorticoid dose among treated patients showed limited variation throughout the year. The Fluctuations observed were attributable to intraarticular and intravenous injections, which involve higher dose administered on a single day, rather than a continuous treatment.

Overall, the proportion of glucocorticoid-exposed patients was high, 428 (75%), substantially higher than previously reported in other Danish studies [4, 6], however quite in line with values reported in the QUEST-RA study, and the study by Moore and Wallace 2021 [4, 5]. This highlights the importance of the chosen index date and lookback window, as well as the inclusion of intraarticular glucocorticoid injections when defining glucocorticoid exposure, as many patients with RA will receive glucocorticoid therapy at some stage during their disease course. Not surprisingly Table 1 illustrates a high correlation between DAS28-CRP and the use of glucocorticoid therapy, with patients with higher DAS28-CRP also receiving a higher average glucocorticoid dose during the first 21 days after first visit.

As treatment initiation in the clinic are expected to occur close to or on first visit for most patients, it was not surprising that the day with the highest number of patients receiving glucocorticoid therapy coincided with the date of first visit, 313 (55%). This number was in line with report on the Danish population in the QUEST-RA study [4], although it remains roughly double the proportion reported by Soussi et al. [6]. The difference likely reflects variation in index date definitions and exposure classification. In contrast to studies relying on prescription-based exposure measures and predefined exposure windows, the present study used the first registered rheumatology visit as index date and assessed physician-prescribed glucocorticoid use based on medical record review and including intraarticular glucocorticoid injections. Figure 2 shows that the average daily dose was substantially reduced after the first few days, with a few large fluctuations for high administered injection doses. This indicates that despite the high proportion of glucocorticoid users, clinicians generally taper treatment effectively during the first year.

Secondarily, this study found that the estimated risk of infections requiring treatment with antibiotics was slightly higher for individuals receiving initial glucocorticoid treatment the first 21 days after the initial visit compared to nonexposed (Table 2). However, the wide CI, indicate that the study may have been underpowered to detect modest differences in infection risk. Although point estimates indicated a higher risk among glucocorticoid-exposed patients, adjustment for comorbidities such as asthma or COPD together with sex and age reduced the RR compared to nonexposed but did not change overall conclusions. In this context, our findings are compatible with results from the GLORIA trial, which reported a favourable benefit–risk balance for low-dose glucocorticoid treatment [9]. At the same time, the direction of the estimates is also consistent with observational studies reporting an increased infection risk with higher glucocorticoid doses [8, 10, 17]. Taken together, these results suggest that while glucocorticoid may be associated with an increased infection risk, the use of low-dose glucocorticoids, rapid tapering, and intraarticular injections—approaches used in this study—may mitigate these risks. Further investigation with larger studies is warranted to fully assess these factors, as this cohort was not large enough to permit dose-dependent, injection-only, or oral-only analyses; therefore, we cannot rule out differences in bioavailability and duration of action regarding the risk of systemic infection.

The weakness of this study included potential bias by indication together with potential unmeasured confounding, such as methotrexate and other immunosuppressive medication, diabetes mellitus, kidney disease, smoking, body mass index (BMI) and other variables that may affect both infection risk and glucocorticoid use. When interpretating association between glucocorticoid and the infection risk, this should be kept in mind. Bias by indication can occur since both disease activity and glucocorticoid can increase the infection risk, and patients with high disease activity often receive higher glucocorticoid dosage (also seen in Table 1). To accommodate this, we included analysis adjusted for DAS28-CRP. For estimating initial glucocorticoid dose, the first 21 days after first visit was used, and all patients with an infection between first visit and 21 days after first visit was removed in the dose-dependent analysis. This was done to ensure sufficient time to estimate initial glucocorticoid dose, as most patients would initiate treatment at first visit or shortly hereafter. Patients with an infection in this interval was removed to ensure that any registration of infection after the 21 days was not related to the same prior infection. Results in Table 2, suggests that the exclusion of patients with infection within the first 21 days has minimal influence on incidence rates. For transferability, one should keep in mind that though patients within Denmark registered in DANBIO are expected to be rather similar, transferability to other regions might depend on differences in registration, and physician treatment preferences. For countries with different treatment guidelines and population differences, this should be kept in mind before transferring results directly.

An additional consideration one should keep in mind, when interpretating the dose-dependent infection risk is, that patients receiving glucocorticoids at index date are likely to receive glucocorticoids at least through part of the follow-up. This is due to the fact, that glucocorticoids are usually tapered, and does not stop from one day to the next. Thus, some dependence between glucocorticoid use at index date and after index date would be expected, possibly creating unwanted conditioning on the future. Unfortunately, we cannot completely remove this dependency due to the nature of the therapy. On a final note, glucocorticoid dose was recalculated to prednisolone equivalent dosage for easier dose comparison. However, since different administration forms or type of glucocorticoid may have different systemic effects, this should be kept in mind when comparing estimates with other studies using different definitions for exposure groups. Stratifying analysis or adjusting for administration forms would be an interesting analysis to perform, unfortunately we did not have sufficient power to include this in the current study.

For data collection, one should note, that only electronic hospital records and DANBIO was used to identify infections. This meant that any records from primary care, and not registered in hospital records, was not available in this study. Further, we did not consider if patients received prophylactic measures.

The strength of the study was the detailed information obtained on glucocorticoid dose, available from DANBIO and patient hospital records. As a description of the glucocorticoid dose was the primary aim of this study, the high quality of registration together with the high proportion of glucocorticoid-exposed patients allowed for reliable estimation of prescribed glucocorticoid doses during the first year. For further studies, linkage with information on other immunosuppressive therapies may help to identify whether specific patient groups are more likely to receive glucocorticoid treatment.

The fact that 24% of patients were excluded in the sensitivity analysis indicates that in DANBIO the time between first visit and diagnosis, may still differ substantially. This should be considered when interpretating studies that use first visit as index date, despite the high positive predictive value of DANBIO [13, 14]. Fortunately, our findings did not show major differences between the full population and the sensitivity analysis. This may suggest that, despite differences in time since diagnosis the included patients may have other attributes that makes them comparable.

## Conclusion

In conclusion, physicians treated a high proportion of patients with glucocorticoid during the first year after the initial visit, with the average dose decreasing substantially after the initial visit. The percentage of glucocorticoid-exposed individuals one year after treatment initiation was markedly reduced, suggesting that physicians in the Danish healthcare decrease glucocorticoid dose within the first year after diagnosis.

Although the incidence rate of infections requiring treatment with antibiotics was higher among patients receiving initial glucocorticoid therapy, the CI for the RR of infections were so wide they were compatible with both harm, benefit and no effect.

## Supplementary Information

Below is the link to the electronic supplementary material.

Supplementary Material 1
