# Supplementary material for: Glucocorticoid use among patients with rheumatoid arthritis during the first year of treatment—a cohort study
Source: Rheumatol Int. 2026 Jun 12;46(7):144. doi: 10.1007/s00296-026-06186-1 (PMC13263268; doi:10.1007/s00296-026-06186-1)
Supplement: Supplementary file 1 — Supplementary Material 1 [file 296_2026_6186_MOESM1_ESM.docx]

| Content | |
| --- | --- |
| **Supplementary A** | Supplementary Results |
| **Supplementary B** | List of Glucocorticoid medication |

**Supplementary A: Supplementary Results**

| **Table S1 Patient characteristics for individuals with ≤ 6 months between first visit and diagnose** | | | | | |
| --- | --- | --- | --- | --- | --- |
|  | | **nonexposed (n=115)** | **Low/Medium dose (n=227)** | **High dose (n=76)** | **Total^a^ (n=429)** |
| **Age,** mean (sd) | | 57.8 (14.5) | 61.1 (15.0) | 63.9 (15.9) | 60.9 (15.1) |
| **Sex** (women)**,** n (%) | | 74 (64.3) | 142 (62.6) | 46 (60.5) | 269 (63.7) |
| **DAS28-CRP,** mean (sd) | | 3.8 (1.2) | 4.8 (1.0) | 5.6 (0.9) | 4.7 (1.2) |
|  | missing | 28 | 45 | 20 | 97 |
| **COPD,** n (%) | | 5 (4.3) | 22 (9.7) | 10 (13.2) | 41 (9.5) |
| **Asthma,** n (%) | | 15 (13.0) | 19 (8.4) | 6 (7.9) | 44 (10.3) |
| **Year of first visit,** n (%) | |  |  |  |  |
|  | 2014 to 2016 | 44 (38.3) | 48 (21.1) | 15 (19.7) | 109 (25.4) |
|  | 2017 to 2019 | 40 (34.8) | 82 (36.1) | 24 (31.6) | 151 (35.2) |
|  | 2020 to 2022 | 31 (27.0) | 97 (42.7) | 37 (48.7) | 169 (39.4) |
| ^a^The total population includes all individuals from date of first visit, whereas the groups on dose include the population from 21 days after first visit. Patients with an infection of lack of follow-up within the first 21 days will occur in the total but not in the dose stratification.  Abbreviations: sd: standard deviation, n: number, DAS28-CRP: 28 joint disease activity score with c-reactive protein, COPD: chronic obstructive pulmonary disease | | | | | |

| **Table S2 Number of treated individuals at selected time intervals** | | | | | |
| --- | --- | --- | --- | --- | --- |
| **For full population** | | | | | |
|  | Time since treatment start | 0 days | 1 to 100 days | 101 to 200 days | 200 to 300 days |
|  | Range for number of treated | 313 | 68 to 169 | 51 to 70 | 47 to 57 |
| **For population with ≤ 6 months between first visit and diagnose** | | | | | |
|  | Time since treatment start | 0 days | 1 to 100 days | 101 to 200 days | 200 to 300 days |
|  | Range for number of treated | 266 | 43 to 139 | 28 to 45 | 23 to 33 |

| **Figure S1 Observed daily glucocorticoid dose translated to prednisolone equivalent dose for patients with ≤ 6 months between first visit and diagnose** |
| --- |
| **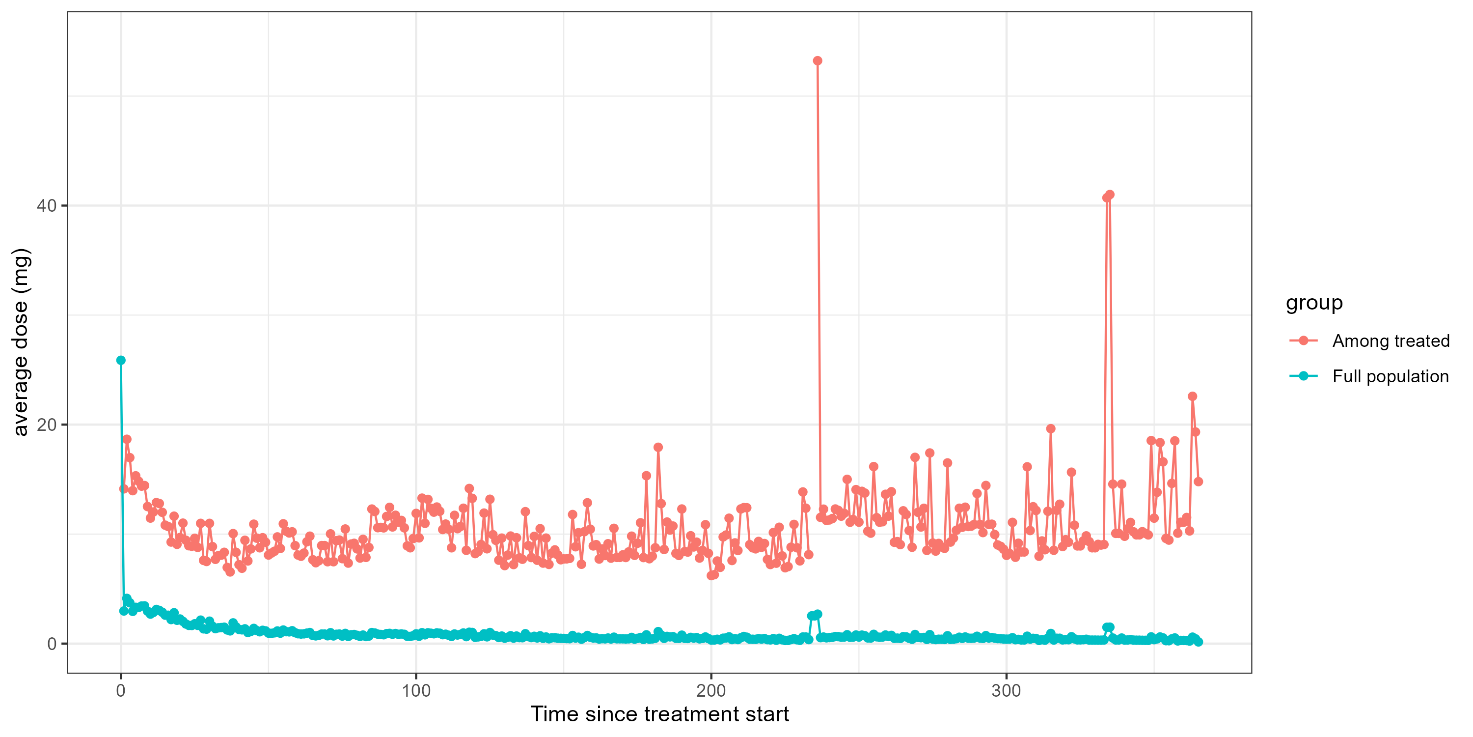** |

| **Tabel S3 Number of patients on different types of glucocorticoid administration.** | | |
| --- | --- | --- |
|  | **Overall (n=574)** | **With ≤ 6 months between first visit and diagnose (n=429)** |
| Oral, n (%) | 262 (45.6) | 211 (49.2) |
| Intraarticular injections, n (%) | 270 (47.0) | 221 (51.5) |
| Intramuscular injections, n (%) | 182 (31.7) | 156 (36.4) |
| Intravenous injections, n (%) | 32 (5.6) | 24 (5.6) |

| **Table S4 Infection risk, for the whole period and stratified on dosage based on first 21 days of treatment for patients with ≤ 6 months between first visit and diagnose.** | | | | | |
| --- | --- | --- | --- | --- | --- |
|  |  | **nonexposed (n=115)** | **Low/Medium dose (n=227)** | **High dose (n=76)** | **Total^a^ (n=429)** |
| **Observed values** | | | | | |
|  | Infections | 19 | 43 | 15 | 88 |
|  | Person years | 104 | 200 | 66 | 373 |
|  | Incidence rate | 0.18 | 0.21 | 0.23 | 0.24 |
| **Estimates from Poisson regression** | | | | | |
|  | Crude RR | 1 (ref.) | 1.21 (0.70 to 1.87) | 1.20 (0.65 to 2.20) | - |
|  | Adjusted RR^b^ | 1 (ref.) | 1.05 (0.65 to 1.7) | 0.99 (0.55 to 1.76) | - |
|  | Complete case disease-activity adjusted RR^c^ |  | 1.32 (0.68 to 2.56) | 1.26 (0.54 to 2.99) | - |
| ^a^The total population includes all individuals from date of first visit, whereas the groups on dose include the population from 21 days after first visit. Patients with an infection of lack of follow-up within the first 21 days will occur in the total but not in the dose stratification.  ^b^The adjusted RR were adjusted for asthma or COPD, sex, and age.  ^c^The complete case adjusted RR were adjusted for asthma or COPD, sex, age, and DAS28-CRP  Abbreviations: RR: relative risk, n: number, DAS28-CRP: 28 joint disease activity score with c-reactive protein, COPD: chronic obstructive pulmonary disease | | | | | |

| **Table S5 RR for infection with binary exposure** | | |
| --- | --- | --- |
|  | **Non-exposed (n=115)** | **Exposed (n=303)** |
| Crude RR | 1 (ref.) | 1.16 (0.72 to 1.86) |
| Adjusted RR^a^ | 1 (ref.) | 1.04 (0.66 to 1.64) |
| Complete case disease-activity adjusted RR^b^ | 1 (ref.) | 1.32 (0.68 to 2.54) |
| ^a^The adjusted RR were adjusted for asthma or COPD, sex, and age.  ^b^The complete case adjusted RR were adjusted for asthma or COPD, sex, age, and DAS28-CRP.  Abbreviations: n: number, DAS28-CRP: 28 joint disease activity score with c-reactive protein, COPD: chronic obstructive pulmonary disease, RR: relative risk, ref: reference. | | |

**Supplementary B: List of Glucocorticoid medication**

The list below contains all glucocorticoid medications included as exposure. Steroid cream and inhalation medications are not included where not included in the study.

| **List of active drugs noted in registered glucocorticoid therapy** |
| --- |
| Betamethasone, Dexamethasone, Fluocortolone, Methylprednisolone, Paramethasone, Prednisolone, Prednisone, Triamcinolone, Hydrocortisone, Cortisone, Prednylidene, Rimexolone, Deflazacort, Cloprednol, Meprednisopne, cortivazol |
